# Differences in mitochondrial function and morphology during cooling and rewarming between hibernator and non-hibernator derived kidney epithelial cells

**DOI:** 10.1038/s41598-017-15606-z

**Published:** 2017-11-14

**Authors:** Koen D. W. Hendriks, Eleonora Lupi, Maarten C. Hardenberg, Femke Hoogstra-Berends, Leo E. Deelman, Robert H. Henning

**Affiliations:** Department of Clinical Pharmacy and Pharmacology, University Medical Centre Groningen, University of Groningen, Hanzeplein 1, 9713 GZ Groningen, The Netherlands

## Abstract

Hibernators show superior resistance to ischemia and hypothermia, also outside the hibernation season. Therefore, hibernation is a promising strategy to decrease cellular damage in a variety of fields, such as organ transplantation. Here, we explored the role of mitochondria herein, by comparing epithelial cell lines from a hibernator (hamster kidney cells, HaK) and a non-hibernator (human embryonic kidney cells, HEK293) during cold preservation at 4 °C and rewarming. Cell survival (Neutral Red), ATP and MDA levels, mitochondrial membrane potential (MMP), mitochondrial morphology (using fluorescent probes) and metabolism (seahorse XF) were assessed. Hypothermia induced dispersion of the tubular mitochondrial network, a loss of MMP, increased oxygen radical (MDA) and decreased ATP production in HEK293. In contrast, HaK maintained MMP and ATP production without an increase in oxygen radicals during cooling and rewarming, resulting in superior cell survival compared to HEK293. Further, normothermic HaK showed a dispersed mitochondrial network and higher respiratory and glycolysis capacity compared to HEK293. Disclosing the mechanisms that hibernators use to counteract cell death in hypothermic and ischemic circumstances may help to eventually improve organ preservation in a variety of fields, including organ transplantation.

## Introduction

Hibernating species have the remarkable ability to preserve their organs in the face of gross and rapid physiological changes during repetitive hibernation phases^1–3^. Hibernation consists of torpor phases, characterized by low metabolism, low body temperature and hypoxia, which are alternated with interbout arousals consisting of shorter periods of rapid restoration of metabolism, reperfusion and normalization of body temperature^4,5^. Although renal blood flow drops with 90% during torpor, glomerular architecture remains preserved^6^. Remarkably, outside the hibernating season, hibernators are also resistant to ischemia and reperfusion (I/R) injury in various organs^7–10^, suggesting adaptations at the cellular level. Indeed, we previously found hamster cells to be resistant to cooling and rewarming injury because of increased H_2_S production through upregulation of the enzyme cystathionine-β-synthase (CBS)^11^. Combining these features, hibernating animals are an optimal natural model to study for new preservation techniques in a variety of fields, such as organ transplantation^12^.

Mitochondria fulfil an important and well-known role in cell survival in I/R, either promoting survival by producing ATP to fuel cellular processes, or inducing cell damage and death by generating reactive oxygen species (ROS) and initiating apoptosis^13^. Mitochondria can produce ATP by oxidative phosphorylation, using the electron transport chain to generate a mitochondrial membrane potential (MMP) that fuels ATP synthase to generate ATP. ATP can also be produced in an anaerobic way by glycolysis, by converting glucose to pyruvate and eventually lactate. Further, mitochondrial function is related to the morphology of the mitochondrial network, ranging from numerous small individual organelles to a single large hyperconnected tubular network, depending on environmental conditions^14–16^. In this network, fission of mitochondria serves to remove damaged mitochondria with or without dysfunctional mitochondrial DNA (mtDNA) by directing them towards degradation by a special form of autophagy (i.e. mitophagy), ultimately resulting in the pruning of dysfunctional branches of the mitochondrial network^17,18^. In contrast, mitochondrial fusion allows efficient mixing and transfer of mitochondrial metabolites, enzymes and mitochondrial gene products throughout the entire mitochondrial unit, thus supplying damaged or less functional mitochondria. Therefore, a strongly connected mitochondrial network is believed to optimize mitochondrial respiratory function^19^.

Given the resistance of hibernators to hypothermia independently of the season, we hypothesized their cells to have mitochondrial adaptations conferring resistance to cell stress. To investigate this, we explored differences between epithelial kidney cells from a hibernating species (Syrian Hamster Kidney cells, HaK) and human kidney cells (HEK293) in survival, mitochondrial morphology and function throughout hypothermia and rewarming. Understanding the mechanisms that hibernators use to overcome cell death in hypothermic or ischemic circumstances may help to eventually improve organ preservation in a variety of fields, including organ transplantation.

## Results

### Hamster kidney cells are resistant to cold-induced stress compared to human kidney cells

**Figure 1:**
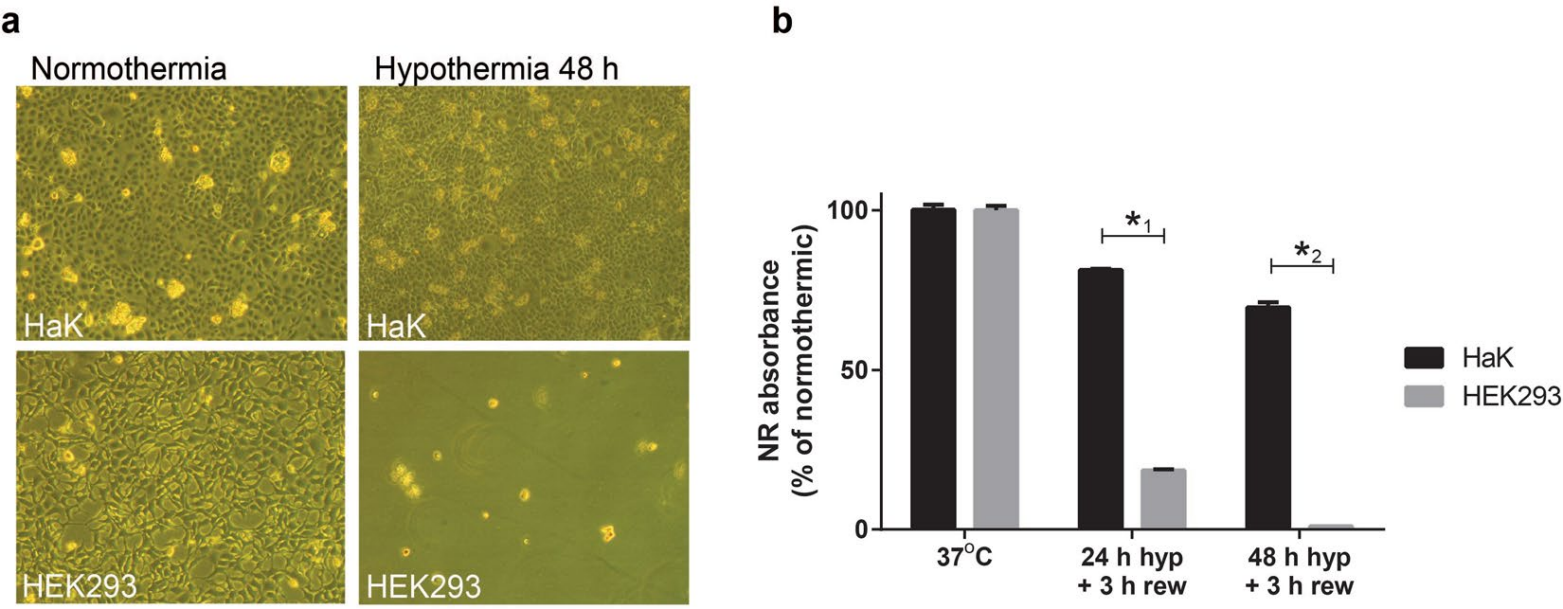
Cell viability after hypothermia followed by rewarming in HaK and HEK293. (**a**) Representative pictures of cell cultures taken in normothermia and immediately after 48 h hypothermia. **(b)** Percentage of neutral red (NR) absorbance of HaK and HEK293 after 24 h and 48 h of hypothermia (hyp) followed by 3 h rewarming (rew). Normothermic NR absorbance was set to 100%. Data presented as mean ± SEM. *1 + *2 p =  < 0.001, t-test, n = 3 per condition).

To determine whether hibernator derived cells are more resistant to hypothermic-induced stress compared to non-hibernator derived cells, effects on viability were assessed in 90% confluent hamster kidney cells (HaK) and human embryonic kidney cells (HEK293) following cold storage for 24 h or 48 h at 4 °C followed by 3 h of rewarming at 37 °C (Fig. 1). Hypothermia and rewarming decreased neutral red (NR) absorbance in 24 h cooled and rewarmed HEK293 by 80% compared to normothermic cells, whereas NR absorbance in HaK decreased by only 20%. Similarly, 48/3 h of hypothermia/rewarming in HEK293 resulted in a NR absorbance below the level of detection, whereas NR absorbance of HaK remained comparable to 24/3 h hypothermia/rewarming at about 70%.

**Figure 2:**
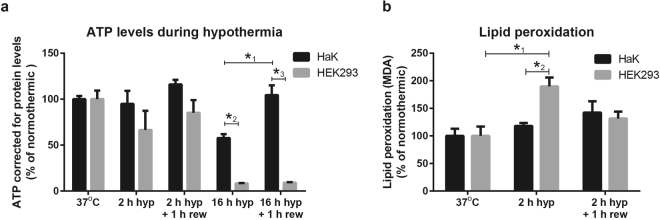
ATP and lipid peroxidation production in HaK and HEK293 throughout hypothermia (hyp) and rewarming (rew). (**a**) Relative ATP levels corrected for the protein concentration where 37 °C control was set to 100%. Data presented as mean ± SEM (5 wells per condition, 2 independent experiments, *1 p = 0.001, one way ANOVA Bonferroni’s post hoc to all 5 conditions of HaK. *2, *3 p < 0.001, t-test. (**b**) Relative lipid peroxidation measured in malondialdehyde (MDA) corrected for to protein concentration where 37 °C control was set to 100%. Data presented as mean ± SEM (5 wells per condition, 2 independent experiments, *1 p = 0.004, one way ANOVA Bonferroni’s post hoc to all 3 conditions of HEK293. *2, p = 0.003, t-test).

Next, we examined differences in energy reserves during hypothermia and rewarming by quantifying ATP levels in both HaK and HEK293 at 2 and 16 h of hypothermia with or without 1 h of rewarming. ATP levels were expressed relative to protein concentration to correct for the observed cell death. Two hours of hypothermia both with and without rewarming did not significantly affect ATP levels in both HaK and HEK293 (Fig. 2a), albeit HEK293 showed a trend towards reduction which restored after an additional 1 h of rewarming. Hypothermia for 16 h with and without rewarming strongly reduced ATP levels in HEK293 to about 8% of normal normothermic values (Fig. 2a). In contrast, 16 h of hypothermia induced a far smaller drop in ATP levels in HaK, still amounting of 60% of normal, which restored to 100% following subsequent rewarming (Fig. 2a).

Since HEK293 apparently fail to maintain ATP production during hypothermic storage – whereas HaK do -, we next examined reactive oxygen species (ROS) production as sign of mitochondrial malfunction by quantifying lipid peroxidation (Fig. 2b). Interestingly, lipid peroxidation markedly increased in HEK293 after 2 h of hypothermia, which normalized following an additional 1 h of rewarming. In contrast, lipid peroxidation levels were unaffected in HaK by 2 h of hypothermia with and without rewarming.

### Oxygen consumption, glycolysis and mitochondrial membrane potential in hypothermia and rewarming

**Figure 3:**
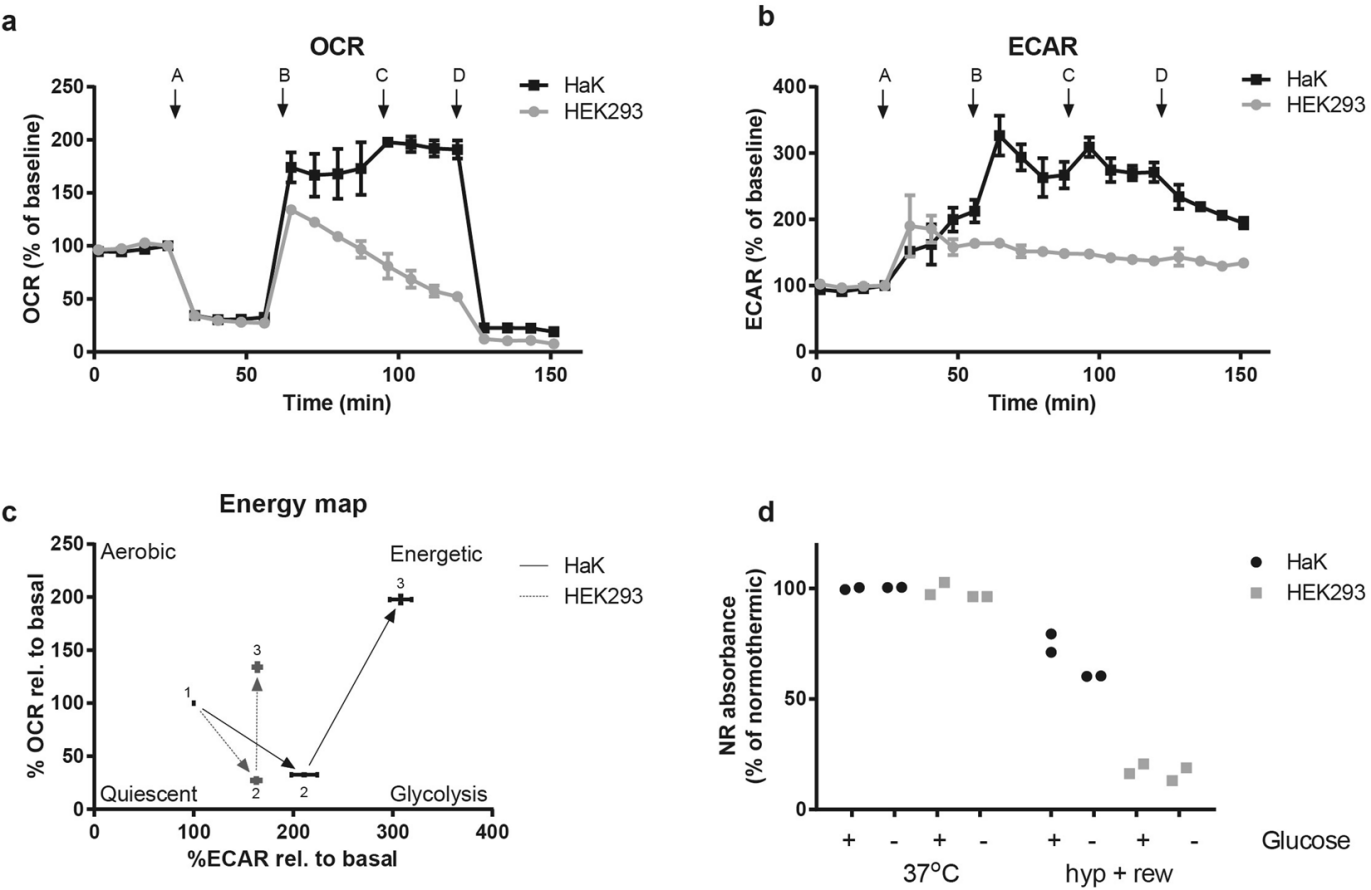
Functional differences of mitochondria between HaK and HEK293. (**b**) Oxygen consumption rate (OCR) relative to basal values of HaK and HEK293 in time (minutes). First basal values, A: oligomycin administration (0.5 µM), B: FCCP administration (0.5 µM), C second FCCP administration (1.0 µM), D: rotenone + antimycin A mix administration (1.0 µM). Data presented as mean ± SEM, n = 3. (**b**) Extracellular acidification rate (ECAR) relative to basal values of HaK and HEK293 in time (minutes). First basal values, A: oligomycin administration (0.5 µM), B: FCCP administration (0.5 µM), C second FCCP administration (1.0 µM), D: rotenone + antimycin A mix administration (1.0 µM). Data presented as mean ± SEM, n = 3. (**c**) Energy map: fist value: basal conditions, second value: blocked complex IV by oligomycin administration, third value: uncoupled membrane potential by FCCP administration. Data presented as relative to basal values where basal value was set to 100%. Data presented as mean ± SEM, n = 3 (**d**) Cell viability after 16 h hypothermia (hyp) + 1 h rewarming (rew) with and without glucose in HaK and HEK293, measured as NR absorbance. Data presented as a dot plot with 2 samples per condition.

To obtain insight into functional differences between HaK and HEK293 mitochondria, we first mapped mitochondrial function by quantifying oxygen consumption rate (OCR) and extracellular acidification rate (ECAR) using Seahorse® XF extracellular flux analyser technology at 37 °C (Fig. 3a–c). Basal OCR and ECAR were measured and set to 100%. Blockage of ATP synthase with oligomycin resulted in a similar drop in OCR to 30% of baseline in both HaK and HEK293. As expected, addition of the mitochondrial membrane uncoupler FCCP, resulted in an increased OCR in both cell lines. Interestingly, OCR increased to 130% of basal levels in HEK293, whereas HaK showed a significantly more prominent increase to 170% of basal levels (n = 3, p = 0.04, Student’s t-test) ECAR values increased in both cell lines after inhibition of ATP synthase (complex V), however, HaK showed a significantly stronger increase in ECAR compared to HEK293 (n = 3, p = 0.03, Student’s t-test). Interestingly, after the mitochondrial membrane potential (MMP) was uncoupled with FFCP, ECAR levels remained stable in HEK293, whereas HaK showed a strong additional increase (n = 3, p = 0.001, Student’s t-test). The exact data without baseline correction of OCR and ECAR is outlined in supplementary Fig. 1.

To verify that the increased ECAR values of HaK resulted from increased glycolysis, the effects of hypothermia and rewarming on cell viability were assessed in HaK and HEK293 cultured in glucose free medium, supplemented with galactose to allow for oxidative phosphorylation. While glucose depletion did not affect cell viability of HEK293 following hypothermia and rewarming, HaK showed a decreased cell viability upon combining hypothermia and glucose depletion (Fig. 3d).

**Figure 4:**
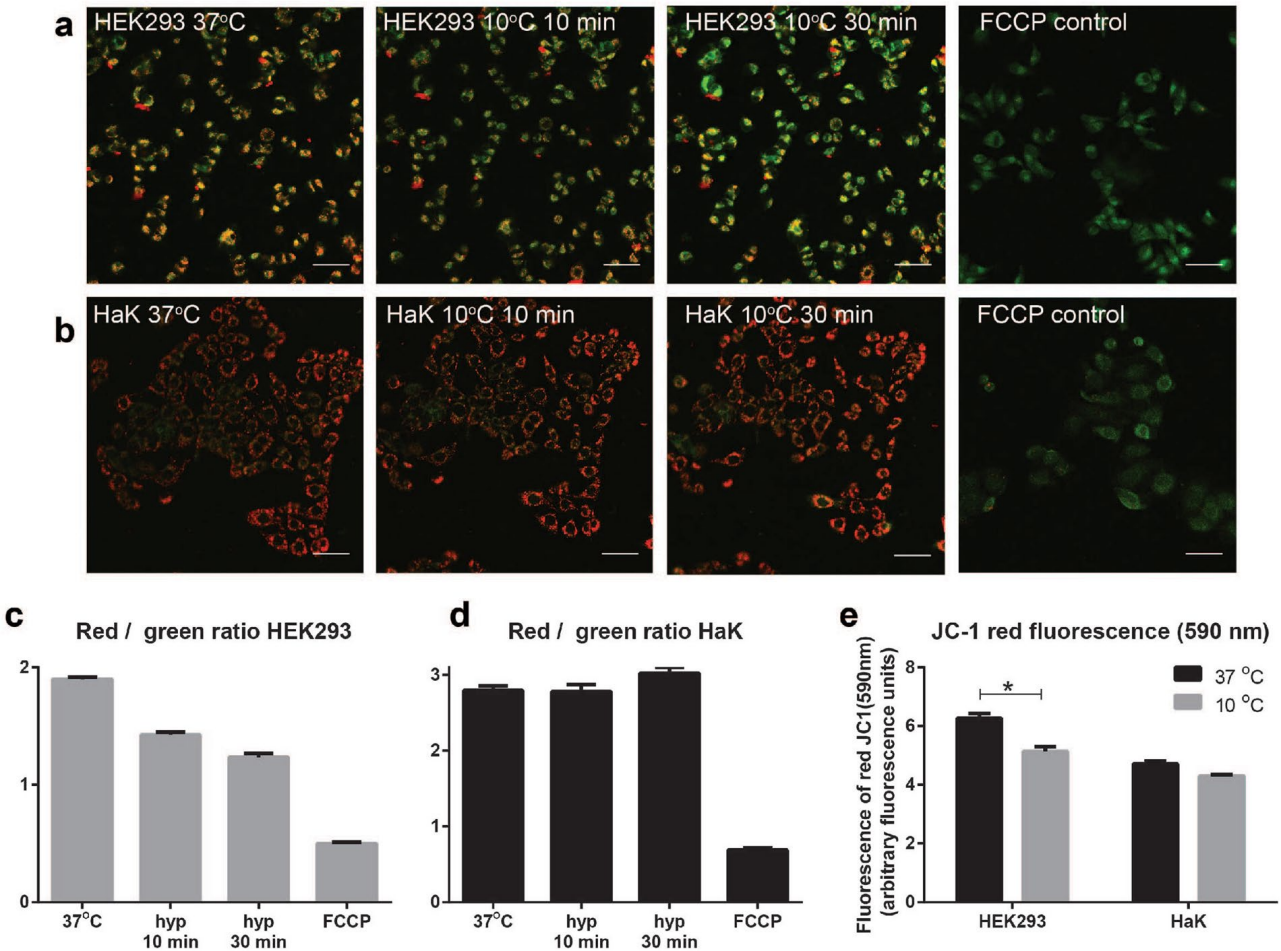
Mitochondrial membrane potential in HaK and HEK293 during early hypothermia. (**a**) JC-1 fluorescence during hypothermia in living HEK293 (40x). To improve visualization brightness was adjusted equally across panel A using ImageJ. Scale bar represent 50 µm. (**b)** JC-1 fluorescence during hypothermia in living HaK. To improve visualization brightness was adjusted equally across panel B using ImageJ. Scale bar represent 50 µm. (**c**) HEK293 red/green emission of pictures in panel A per cell (arbitrary fluorescence units). (**d**) HaK red/green emission of pictures in panel B per cell (arbitrary fluorescence units). (**e**) MMP measured as red fluorescence JC-1 (590 nm) in normothermic and 30 min hypothermic HaK and HEK293 (arbitrary fluorescence units). Data presented as mean ± SEM (n = 7 per condition p < 0.001, student’s t test).

Next, we explored the mitochondrial membrane potential (MMP) following induction of hypothermia using the fluorescent dye JC-1, of which the emission spectrum shifts from red to green upon loss of MMP. Gradual cooling of HEK293 from 37 °C to 10 °C in 10 min resulted in a substantial reduction in red/green ratio of HEK293 which decreased even more after 30 min of hypothermia (Fig. 4a,c). In contrast, the red/green ratio of HaK remained unchanged after exposure to hypothermia (Fig. 4b,d), indicating that the MMP of HaK was maintained during the 30 min of cooling. Functionality of JC-1 was confirmed both in HEK293 and HaK by loss of MMP upon addition of FCCP (Fig. 4a,b). Since laser power and gain was set per channel per cell line, the ratios cannot be compared directly between the two cell lines. The loss of total fluorescence of the red signal (590 nm) in normothermic compared to 30 min hypothermic HEK293 and HaK is displayed in Fig. 4e. To control for bleaching effects, HaK and HEK293 cells were exposed to the same protocol as described above. As shown in supplementary Fig. 2, bleaching effects were equal in the red and green channel.

### Mitochondrial network structure during hypothermia

**Figure 5:**
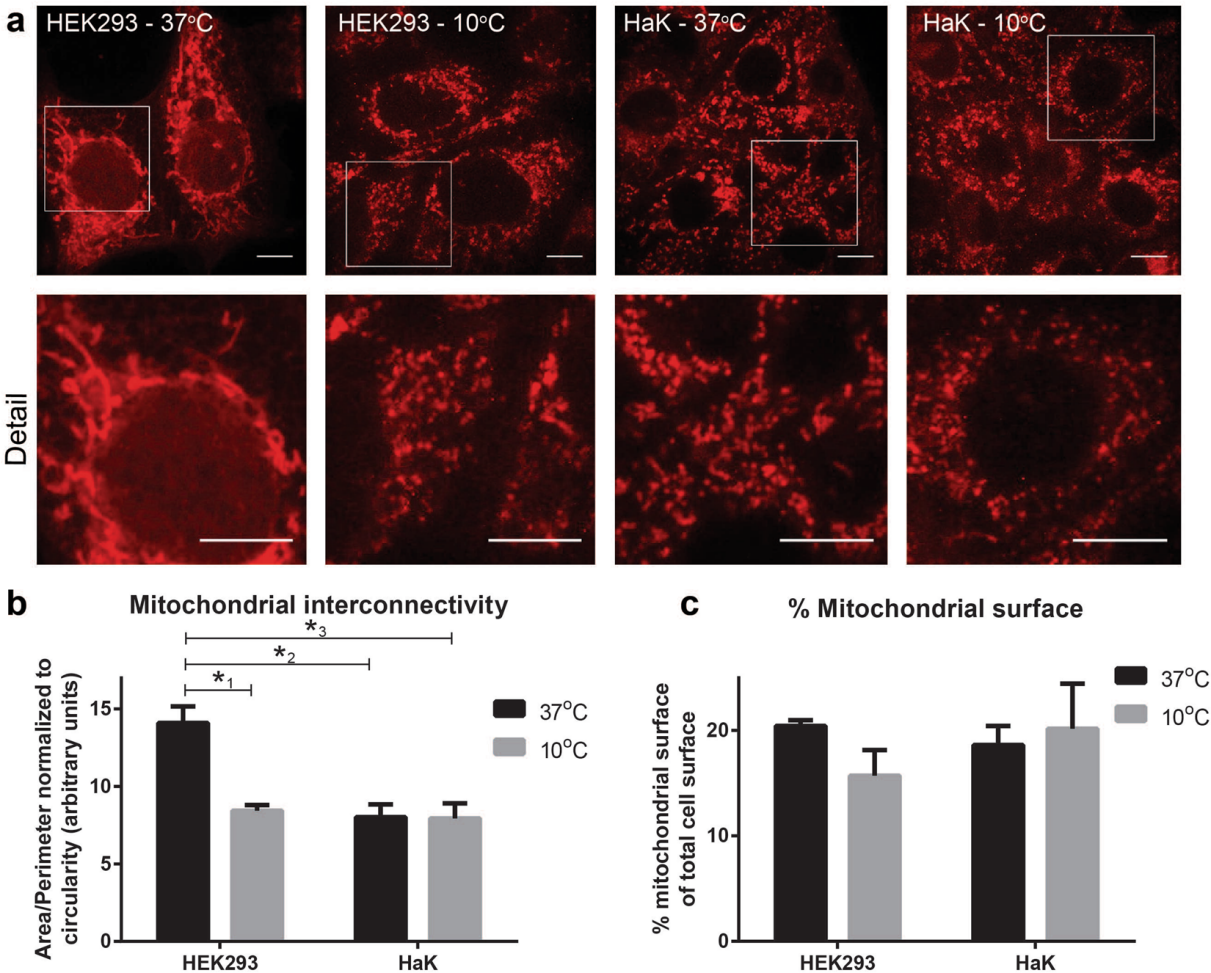
Mitochondrial morphology and interconnectivity in HaK and HEK293 during early hypothermia. (**a**) Typical examples of the mitochondrial morphology in normothermic and hypothermic (24 h 10 °C) HEK293 and HaK as visualized by MitoTracker staining (95x). To improve visualisation brightness was adjusted using ImageJ. Scale bar represent 10 µm. (**b**) Mitochondrial interconnectivity expressed by mitochondrial area/mitochondrial perimeter. Data presented as mean ± SEM (n = 5 per condition, *1 p < 0.001, *2 p = 0.001, *3 p = 0.001, ANOVA Bonferroni’s post hoc) (**c**) Percentage of mitochondrial content of total cell surface. Data presented as mean ± SEM (n = 5 per condition).

As proper mitochondrial function depends on patency of the mitochondrial network, we next visualized the effect of hypothermia using the MitoTracker fluorescence dye for living cells. Whereas normothermic HEK293 showed a highly-connected network consisting of elongated and slim mitochondria, hypothermia induced a total dispersion of this network (Fig. 5a). In contrast, both normothermic and hypothermic HaK displayed a similar and dispersed mitochondrial network consisting of smaller mitochondrial particles. Mitochondrial interconnectivity was calculated by dividing the mitochondrial area by the mitochondrial perimeter (Fig. 5b). In accord with the microscopic images, a significant higher interconnectivity was found in normothermic HEK293 compared to hypothermic HEK293 (n = 5 per conditions, p < 0.001, ANOVA), whereas HaK showed a low interconnectivity both in normothermic and hypothermic conditions. Despite the dispersion of the mitochondrial network upon hypothermia, no significant change in total mitochondrial surface area was seen in HEK293 (Fig. 5c), with both cell lines showing a comparable mitochondria surface area amounting about 20% of the cell surface area.

**Figure 6:**
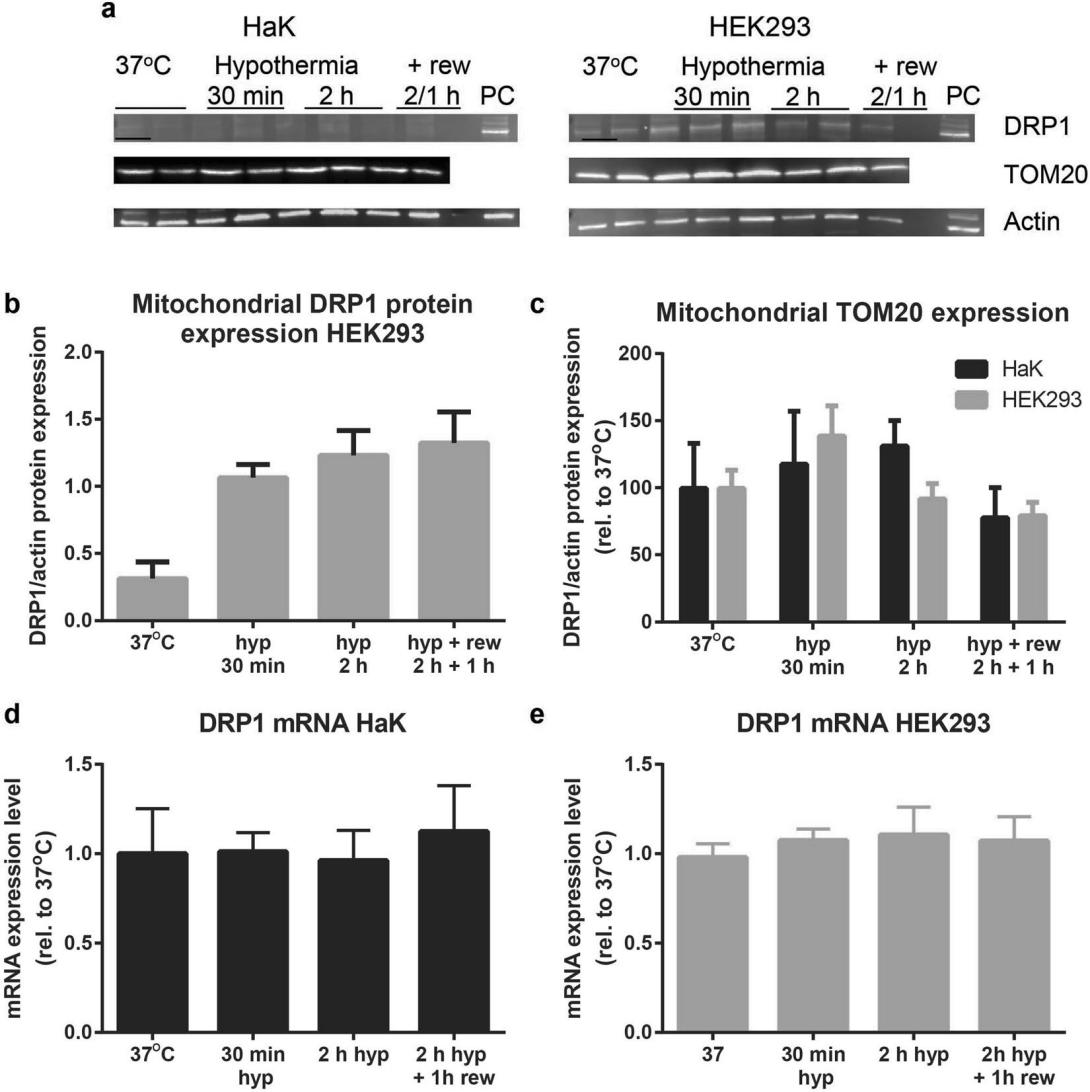
DRP1 and TOM20 expression during hypothermia and rewarming in HaK and HEK293. (**a**) Western blots. PC = positive control (Hek293 whole cell lysate). Full lengths blots are shown in supplement Fig. 3 (**b**) Mitochondrial DRP1 in HEK293 during hypothermia and rewarming. Data presented as mean ± SEM (n = 2 per condition) (**c**)**:** Mitochondrial TOM20 levels throughout hypothermia (hyp) and rewarming (rew) for HaK and HEK293. Data presented as mean ± SEM (n = 2 per condition).(**d**) mRNA levels of DRP1 in HaK during hypothermia and rewarming (n = 3). Data presented as mean ± SEM (**e**) mRNA levels of DRP1 in HEK293 during hypothermia and rewarming (n = 6). Data presented as mean ± SEM.

Since we observed hypothermia in HEK293 to induce dispersion of the mitochondrial network, we next examined mitochondrial expression of the fission factor DRP1 and the mitochondrial marker TOM20 during hypothermia and rewarming. Hypothermia for 30 min and 2 h induced a strong increase in mitochondrial DRP1 protein levels in HEK293 without normalization after 1 h of rewarming (Fig. 6b). Unfortunately, the DRP1 antibody did not detect DRP1 of hamster origin, as no specific signal was found in HaK (Fig. 6a). Further, hypothermia with or without rewarming did not affect TOM20 protein levels in either HEK293 or HaK (Fig. 6c), matching the unchanged mitochondrial surface area as found in Fig. 5c.

As an alternative to Western blot, we assessed DRP1 mRNA levels in both HaK and HEK293 by qRT-PCR. However, as opposed to mitochondrial DRP1 levels in HEK293, both cell lines showed unaltered DRP1 mRNA levels during hypothermia compared to baseline in all conditions (Fig. 6d,e). Likely, these discrepancy between mRNA and mitochondrial protein expression is explained by the main mechanism of activity regulation of DRP1, being its translocation from cytosol to mitochondria upon phosphorylation rather than regulation on the expression level^20^. Given the substantial increase in protein levels of DRP1 upon hypothermia in HEK293, but not mRNA levels, we conclude that PCR is not a working alternative for Western blot to examine DRP1 regulation in HaK.

## Discussion

Our results show that the non-hibernator derived kidney cell line HEK293 is vulnerable to hypothermic stress conditions, resulting in a loss of MMP and ATP production and cell death in an *in vitro* hypothermic model. In contrast, hamster kidney epithelial cells showed good cell viability with maintenance of MMP and ATP production both during hypothermia and after rewarming. Also, mitochondria of HEK293 undergo a dispersion of their tubular mitochondrial network in response to hypothermia, whereas non-cooled HaK already show a dispersed mitochondrial network in normothermic conditions. Further, normothermic HaK show a strong and sustained increase in respiratory and glycolysis capacity in response to the MMP uncoupler FCCP compared to HEK293. This is the first study to document differences in mitochondrial morphology and metabolism between hibernator and non-hibernator cells during hypothermia and rewarming. The mitochondrial adaptations of hibernator cells may help to improve hypothermic storage of human organs during transplant procedures.

Our study shows that HEK293 are far more vulnerable to cell death following hypothermic conditions compared to HaK. Differences in maintaining ATP levels seems to be an important reason. Although a 2 h hypothermic period did not significantly decrease ATP levels in our experiments, we showed that HEK293 were unable to maintain ATP levels during 16 h of hypothermia, suggesting that under hypothermic conditions, ATP consumption exceeds ATP production. Although 16 h of hypothermia in HaK also lowered ATP, it was still maintained at an about a 10 times higher level compared to HEK293. Apparently, HaK are superior to HEK293 in balancing ATP production and consumption in hypothermic conditions, as earlier proposed^21^. Only few studies explored mitochondria and cooling in these cell types, particularly HEK293. The strong decrease of ATP content of HEK293 upon hypothermia is in line with previous observations^22^, however was only found at a later time point in our study. Further, Park *et al*.^23^ report MMP in HEK293 to be unaffected by hypothermia at 10 °C up to 30 min. The reason for these discrepancies is unknown, but may be rooted in differences in cell origin, culture media or antibiotics used. Moreover, rate of cooling was not reported in these papers. Such an explanation seems likely also in view of the clear internal consistency of our data on cell death, MMP, and ATP and ROS production.

The difference between HEK293 and HaK to maintain ATP levels under hypothermic circumstances seems related to the spare oxidative capacity, given the stronger and sustained increase in oxygen consumption that euthermic HaK showed after FCCP stimulation compared to HEK293. This stronger increase in OCR levels suggest that HaK have a more potent oxidative phosphorylation compared to HEK293. HaK also showed an increased ECAR after inhibition of ATP synthase and FCCP administration, suggesting the cell to deploy a potent activation of anaerobic ATP production via glycolysis, potentially functioning as back-up system to secure ATP synthesis. In agreement, preclusion of glycolysis by glucose depletion prior to hypothermia and rewarming markedly decreased cell survival in HaK. This adaptation to anaerobic ATP production is in accord with the observation that the availability of non-lipid metabolic fuels relates to torpor duration in hibernating mammals^24^. As reviewed by Perry *et al*.^25^, usage of JC-1 as indicator of the MMP is subject to technical pitfalls such as bleaching, equilibration time and surface/volume changes. However, these were controlled for in our experiments by control experiments using FCCP and non-cooled cells, showing a fall in the red/green ratio upon a loss of MMP and a stable red/green ratio over time. Moreover, our JC-1 data are completely in line with the changes in ATP levels. Therefore, we conclude that our JC-1 measurements correctly reflect changes in MMP.

The preservation of MMP under hypothermic conditions may constitute another important factor in maintaining of ATP production in HaK. However, in contrast to HaK, HEK293 show a very rapid loss in MMP during 30 min hypothermia. Another contributing factor may be the difference in mitochondrial network structure of HaK and HEK293. In contrast to HaK, whose mitochondria display a dispersed network at all temperatures, normothermic HEK293 have a well-connected tubular mitochondrial network, that fragmented into an extensively dispersed network under hypothermic conditions. Similar to the dispersion of the mitochondrial network in HEK293, dispersion was shown previously in an *in vivo* hypoxic-stress rat model^26^ and a hypothermic^21^ and oxygen/glucose depletion^26^ cell model. A hyperconnected network is thought to support mitochondrial function by allowing for (re)distribution of mtDNA, mitochondrial components and metabolites^18,19^. In accord, pharmacologically fusing the mitochondrial network after cardiac arrest in mice enhanced myocardial performance after cardiac arrest^27^. However, tau induced mitochondrial hyper-fusion is suggested to decrease mitochondrial function and thereby cell viability^28^. It is still unclear how to interpret these differences in network morphology between HEK293 and HaK. Dispersion of the network upon hypothermia in HEK293 may also be viewed as a futile, cell stress-related adaptation to prune damaged mitochondria from the network to limit production of ROS and invoke mitophagy^13,15–19^. Alternatively, the constant dispersion of the mitochondrial network in HaK may be interpreted as an adaptation to deal with hypothermic stress to safeguard mitochondrial function during hibernation. Indeed, pharmacological inhibition of fission during stress is shown to aggravate cell damage^29^. Consequently, differences in mitochondrial function in HaK compared to HEK293 may result from an adaptation to the constantly dispersed nature of a mitochondrial network. Such adaptations in HaK may constitute also a highly efficient mitophagy of damaged mitochondria and/or superior biosynthesis, in turn preserving functional mitochondria and preventing DNA damage.In addition to routing mitochondria towards mitophagy, fission may serve another purpose. Some investigators previously proposed mitochondrial morphology as mechanism to regulate oxygen consumption^30^. Whereas fission can lower oxygen consumption. However, induction of fission by cooling in HEK293 seems not to result in limiting oxygen consumption, given the increased ROS production that we found. Unfortunately, direct evidence could not be obtained, because Seahorse technology precludes measurements at low temperatures.

Defining the exact mechanisms, however, awaits experimentation in (cells derived from) various hibernating species. Further, pharmacological intervention in several pathways linked to mitochondrial stress and ATP production, like OPA1/DRP1, and manipulation of energy sources are of interest. These manipulations may hold potential as cell protection strategies, with the ultimate aim to limit the impact of stress conditions, including cold storage techniques as commonly used in organ transplantation.

## Materials and Methods

### Cell culture

Two kidney epithelial cell lines were used, i.e. HaK (ATCC CCL-15) from the Syrian hamster (hibernator) and HEK293 (ATCC CRL-1573) from human embryonic kidney (non-hibernator). For all experiments cells were grown on 0.001% poly-l-lysine coated surfaces. HaK were cultured in EMEM supplemented with 10% Fetal Bovine Serum (FBS), 1% penicillin/streptomycin (PS) and 1% minimal essential amino acids. HEK293 were cultured in DMEM with 4500 mg/L glucose supplemented with 10% FBS and 1% PS.

### Hypothermia, rewarming and cell viability

To perform hypothermia and rewarming experiments, cells were seeded in six-wells plates and after overnight adherence placed at 4 °C in a standard laboratory refrigerator for different time periods, with or without rewarming by reinstitution of standard cell culture conditions (37 °C). Cell viability was assessed by a Neutral Red (NR) assay to quantify the amount of living cells. Following replacement of normal media by NR media (culture media with 5% FBS and 50 mg/ml NR dye, Sigma Aldrich), cells were lysed and absorbance was measured at 450 nm using a Synergy 2 Multi-Mode plate reader (BioTek).

### ATP and lipid peroxidation

Cells were grown in six-wells plates and hypothermia and rewarming was performed. Following addition of EDTA buffer, cell scraping on ice and boiling for 6 min, ATP was measured with a luciferase assay (Promega) with luminescence measured at 590 nm. MDA was measured using the OxiSelect TBARS assay kit (Cell Biolobs), according to the manufacturer’s protocol. ATP and lipid peroxidation levels were expressed as percentage of fluorescence levels corrected for protein levels, where 37 °C was set to 100%.

### Mitochondrial function

Mitochondrial oxygen consumption rate (OCR) and extracellular acidification rate (ECAR) was measured by a mito-stress test protocol on a Seahorse® XF 24 Analyzer (Seahorse Bioscience, Billerica, MA), quantifying changes in extracellular oxygen tension and pH. After measurements of basal OCR and ECAR levels were obtained, cells were cumulatively incubated with 1 mM oligomycin (ATP Synthase inhibitor, Sigma Aldrich), 0.5 and 1 mM carbonyl cyanide 4-(trifluoromethoxy) phenylhydrazone (FCCP, mitochondrial uncoupler, Sigma Aldrich) to measure non-mitochondrial ATP production and maximum OCR production, respectively. Finally, a mixture of 1 mM Rotenone (Sigma Aldrich) and 1 mM Antimycin A (Sigma Aldrich) was added to completely block mitochondrial electron transport.

### Mitochondrial membrane potential and morphology

To perform time lapse recordings during hypothermia and rewarming, a computer driven temperature controlled aluminium cell culture well was constructed with stable temperature management within the range of 5 °C to 37 °C, including controlled temperature shifts. Mitochondrial membrane potential (MMP) was fluorescently measured by quantifying the fluorescence emission shift from JC-1 (Sigma Aldrich) green (529 nm) monomers to red (590 nm) aggregates, using complete loss of MMP in response to FCCP (2 µM) as positive control. Before analysation, samples were incubated for 20 min in 37 °C to make sure an equilibrium in JC-1 aggregates and monomers was formed. A decrease in measured JC-1 aggregation during cooling as a result of dye dilution due to increased mitochondrial surface can be excluded, since Fig. 5C have shown that the mitochondrial surface is stable or increasing. Since JC-1 is very light sensitive, sample preparation was performed in a dark room and laser power and exposure time was as low as possible. To compare the MMP of warm and hypothermic HaK and HEK293, 7 pictures were taken randomly over the sample in warm cells. Thereafter the sample was gradually cooled to 10 °C and after 30 min again 7 pictures were taken randomly over the sample and the red signal (590 nm) was examined, as outlined in Fig. 4e. To clarify the pictures showed in Fig. 4, brightness was adjusted equally across the entire image using ImageJ. Imaging was performed on a Leica SP2 microscope, using a 40x oil objective. Mitochondrial morphology was examined in cells that were placed in hypothermic conditions for 24 hr, afterwards they were incubated with a fluorescent probe selective for living mitochondria (MitoTracker Deep Red FM [Life Technologies, Invitrogen], 100 nM, 5 min, 37 °C). Images were created on a Solamere Nipkow Confocal Live Cell Imaging microscope. Imaging was performed with a 63x glycerol objective at 644/665 (excitation/emission) with an additional 1,5x objective. Data was analysed using a macro on ImageJ^31^, which following marking of cells borders and background subtraction transforms images to binary black/white pictures using automated settings for lighting, contrast and threshold. Then area, perimeter and circularity of all particles within the encircled cells were calculated. These parameters were used to calculate interconnectivity of mitochondria, expressed as the mitochondrial surface area divided by the mitochondrial perimeter. To improve visualization of Fig. 5, brightness was adjusted equally across the entire image using ImageJ.

### Western blotting

Cytosolic and mitochondrial protein fractions were isolated by density gradient centrifugation following extraction of lysates using RIPA lysis buffer (50 mM Tris-Cl pH = 8.0,150 mM NaCl, 1% Igepal Ca 630, 0.5% Sodium Deoxycholate, 1.0% SDS, 0,4% protein inhibitor cocktail, 100 mM sodium orthovanadate, 1 M NaF, 10 mM B-mercapto ethanol). Protein concentration was measured with an Bioad protein assay on a Synergy H4 plate reader. Samples were loaded to 4–20% SDS precasted gels (Biorad TGX gels) and transferred to a nitrocellulose membrane (Biorad). The membranes were blocked with 5% skimmed milk and incubated with primary (O/N at 4 °C) and secondary (1 h at room temperature) and visualised using SuperSignal (Perkin Elmer) on a GeneSnap (Gene Gnome) system and quantified using GeneTools (GeneGnome). Antibodies used were: anti-DRP1 (1:1.000 Abcam), anti-Tom20 (1:1.000 Santa Cruz), anti-B-actin (1:10.000 Santa Cruz), Goat-anti-rabbit-peroxidase (GARPO, 1:000 Dako), rabbit-anti-mouse-peroxidase (RAMPO, 1:000, Dako). Full membranes are shown in the supplement Fig. S2.

### RNA isolation and qRT-PCR

After experimental treatment, cells were lysed and RNA was obtained using trizol (Ambion). The RNA concentration and purity were determined spectrophotometrically by measuring the absorbance at 260 nm. RNA was reversely transcribed into cDNA and rtPCR analysis was employed to quantify mRNA levels. Samples were prepared with GoTaq buffer (Promega), 0.375 μM of primer and template DNA in a total volume of 10 uL. The PCR profile consisted of 15 min at 95 °C, followed by 40 cycles with heating to 95 °C for 15 seconds and cooling to 60 °C for 1 min. All primers were designed with SE Central (4.10) using default settings and criteria. The sequences of the primers are listed in Table 1. All samples were quantified using a calibration curve. Efficiencies for all PCR reactions were between 80 and 100%.

**Table 1:** Primer sequences.

Gene	Forward (5′ → 3′)	Reverse (5′ → 3′)
DNM1 (DRP1) Human	CCG CGG CAT GGA AGA TCT CA	TCT CGA GCA CCG AGC TCT TG
DNM1 (DRP1) Syrian Hamster	GAG GAG ATG GAA CGC ATT GT	CCG ATG TTG TTG ATG GTC AG

### Statistics

OCR and ECAR data were analysed using Wave Desktop 2.2. Statistical analysis was performed using SPSS Statistics 22. Graphs were created with GraphPad Prism 6. Data are expressed as mean ± SEM. Differences between groups were tested using a student’s t test or ANOVA with Bon Ferroni’s post-hoc. Values deviating from the mean over 2 times the standard variation were considered outliers and discarded from analysis. P-values < 0.05 were considered statistically significant.

## Electronic supplementary material

Supplementary information
