# Supplementary material for: Differences in mitochondrial function and morphology during cooling and rewarming between hibernator and non-hibernator derived kidney epithelial cells
Source: Sci Rep. 2017 Nov 14;7:15482. doi: 10.1038/s41598-017-15606-z (PMC5686174; doi:10.1038/s41598-017-15606-z)

# Supplementary information

# Differences in mitochondrial function and morphology during cooling and rewarming between hibernator and non-hibernator derived kidney epithelial cells.

**Koen DW Hendriks^1^*, Eleonora Lupi^1^, Maarten C Hardenberg^1^, Femke Hoogstra-Berends^1^, Leo E Deelman^1^, Robert H Henning^1^**

1: Department of Clinical Pharmacy and Pharmacology, University Medical Centre Groningen, University of Groningen, Hanzeplein 1, 9713 GZ, Groningen, The Netherlands.

September 12, 2017

**Supplementary fig 1, Seahorse data without normalisation**

**
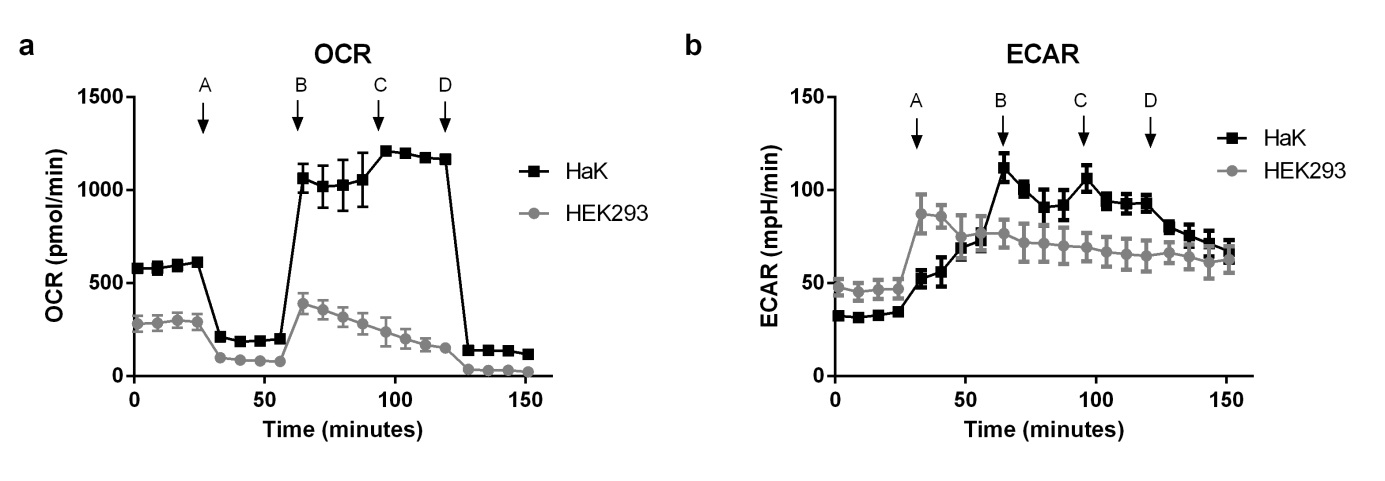

a**: oxygen consumption rate (OCR) values of HaK and HEK293 in time (minutes), expressed as pmol/min. First basal values, A: oligomycin administration (0.5 µM), B: FCCP administration (0.5 µM), C second FCCP administration (1.0 µM), D: rotenone + antimycin A mix administration (1.0 µM). Data presented as mean ± SEM, n=3. **b**: extracellular acidification rate (ECAR) values of HaK and HEK293 in time (minutes), expressed as mpH/min. First basal values, A: oligomycin administration (0.5 µM), B: FCCP administration (0.5 µM), C second FCCP administration (1.0 µM), D: rotenone + antimycin A mix administration (1.0 µM). Data presented as mean ± SEM, n=3.

**Supplementary fig 2, control experiment with JC-1.**


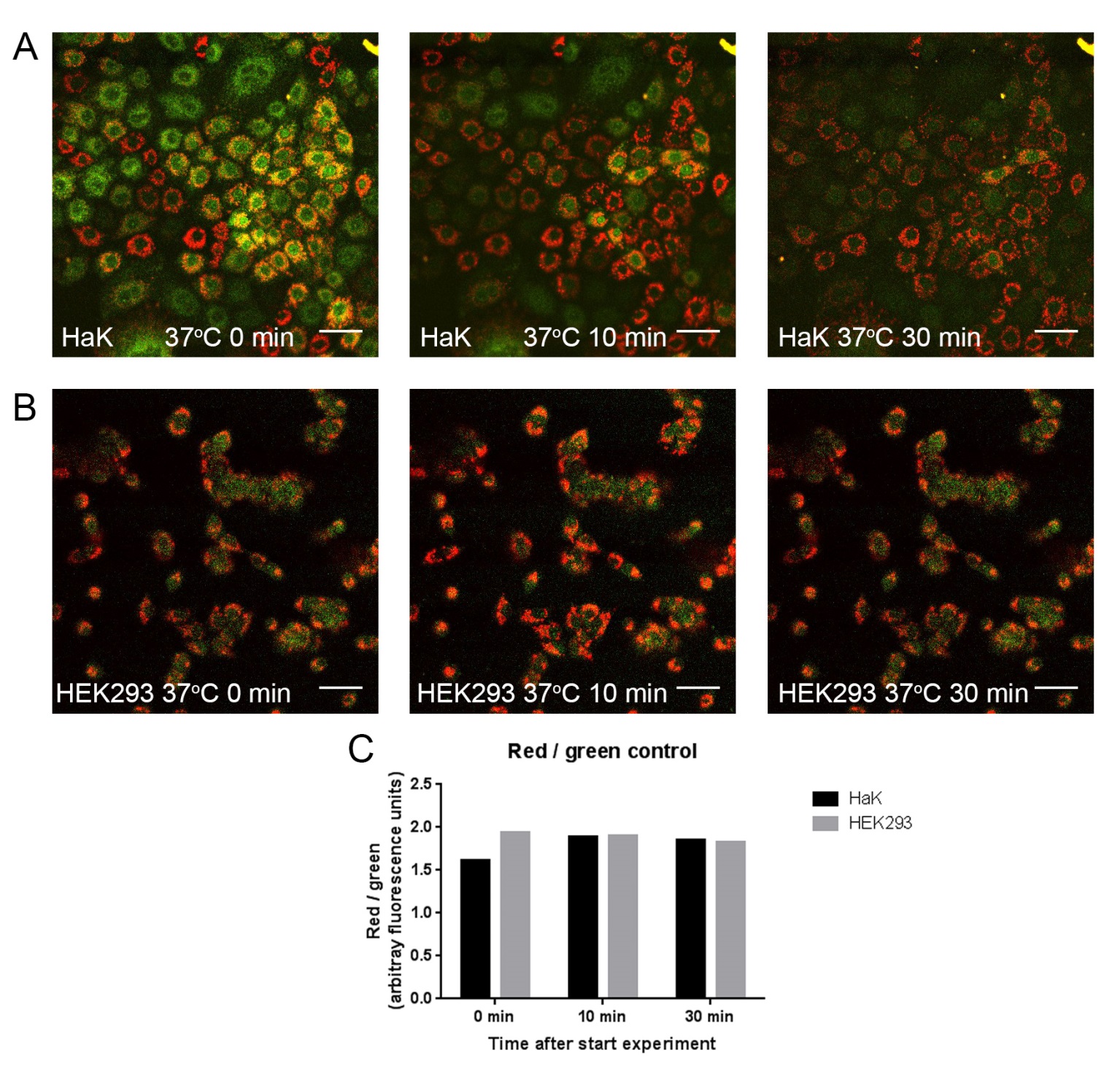


**Supplementary fig 3, full length Western Blots.**

*DRP1 HaK:*

37^o^C Hypothermia + rew
 30 min 2 h 2/1 h PC


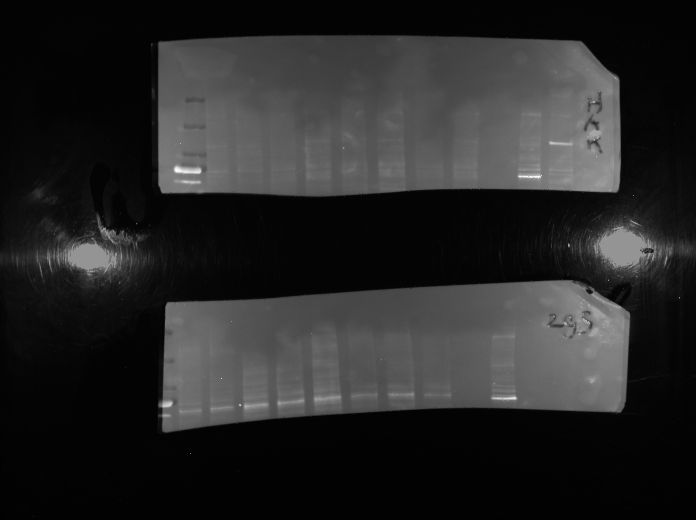


*DRP1 HEK293:*

37^o^C Hypothermia + rew
 30 min 2 h 2/1 h PC


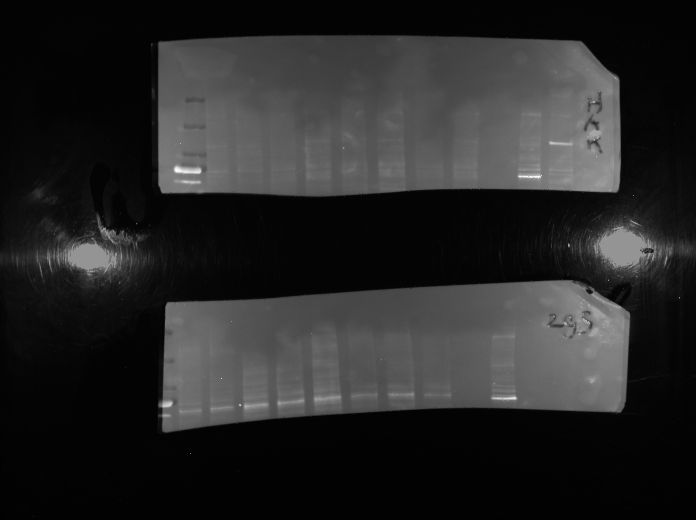


*Tom20 HaK:*

37^o^C Hypothermia + rew
 30 min 2 h 2/1 h


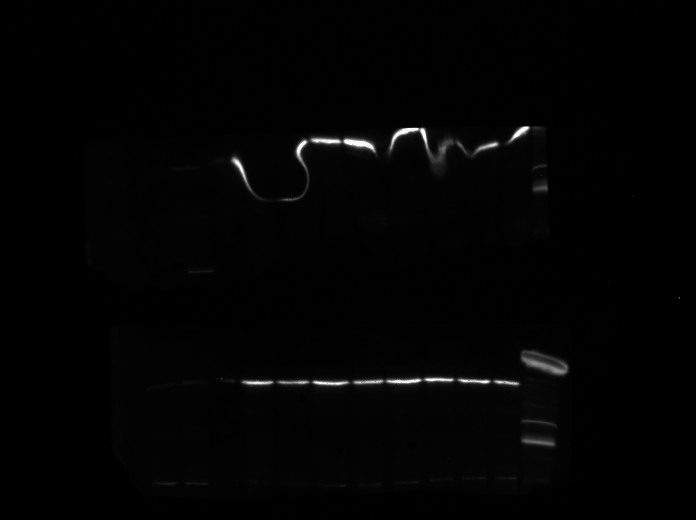


*Tom 20 HEK293:*

37^o^C Hypothermia + rew
 30 min 2 h 2/1 h


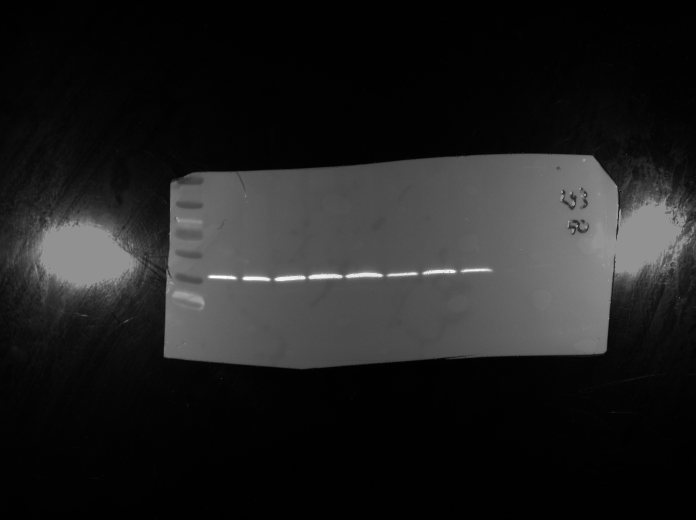


*B-actin HaK*


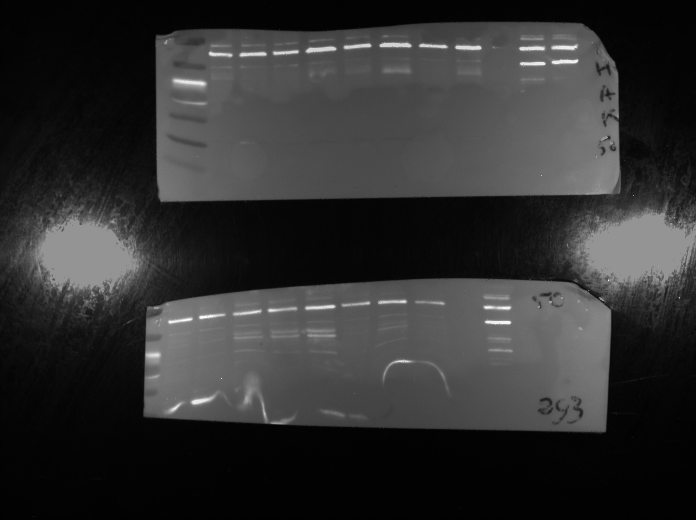


37^o^C Hypothermia + rew
 30 min 2 h 2/1 h PC

*B-actin HEK293*

37^o^C Hypothermia + rew
 30 min 2 h 2/1 h PC


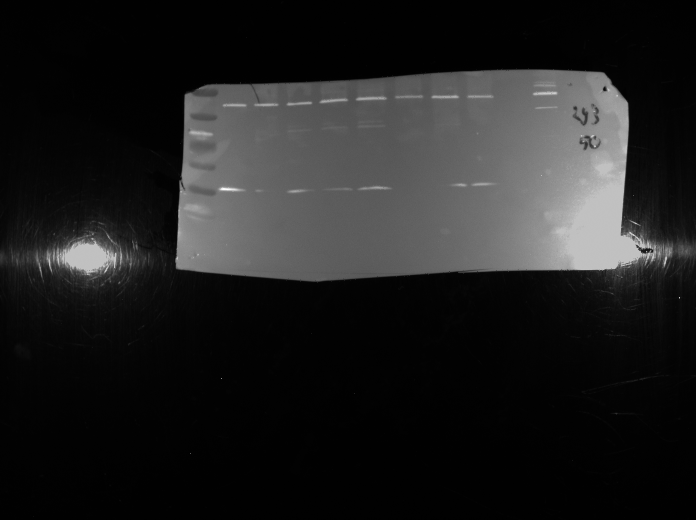

Supplement: Supplementary file 1 — Supplementary information [file 41598_2017_15606_MOESM1_ESM.doc]
